# *Scrophularia striata* Extract Supports Rumen Fermentation and Improves Microbial Diversity *in vitro* Compared to Monensin

**DOI:** 10.3389/fmicb.2018.02164

**Published:** 2018-09-19

**Authors:** Maryam Bagheri Varzaneh, Fenja Klevenhusen, Qendrim Zebeli, Renee Petri

**Affiliations:** ^1^Department of Agriculture, Iranian Research Organization for Science and Technology, Tehran, Iran; ^2^Institute of Animal Nutrition and Functional Plant Compounds, Department for Farm Animals and Veterinary Public Health, University of Veterinary Medicine Vienna, Vienna, Austria

**Keywords:** greenhouse gasses, fermentation profile, phytogenic compounds, ionophores, rumen microbiome, Rusitec

## Abstract

In the search for natural alternatives to antibiotic feed additives, we compared the efficacy of two doses of *Scrophularia striata* extract [*S. striata*-Low at 40 and *S. striata*-High at 80 mg g^-1^ dry matter (DM)] with monensin (monensin) and a negative control in the modulation of rumen fermentation, methane production and microbial abundance *in vitro*. Microbes were investigated using qPCR and 16S rRNA targeted sequencing. Data showed that the addition of *S. striata* increased production of total short chain fatty acids (SCFA) in comparison to both monensin and control (*P* = 0.04). The addition of *S. striata* increased acetate production, and increased propionate at the higher dosage (*P* < 0.001). Supplementation of *S. striata* lowered methane production (*P* < 0.001) compared to control but with no effect compared to monensin. Ammonia concentration decreased by 52% (*P* < 0.001) with *S. striata*-High supplementation (4.14 mmol L^-1^) compared to control, which was greater than that of monensin (36%). The diversity of rumen bacteria was reduced (*P* < 0.001) for monensin and *S. striata* for both the number of observed OTUs and the Chao1 index. Quantitative analysis of Protozoa showed a decrease in the monensin treatment (*P* = 0.05) compared to control. Archaea copy numbers decreased equally in both *S. striata*-High and monensin treatments compared to the control group. Supplementation with *S. striata* increased relative abundances of *Fibrobacteres* (*P* < 0.001) and *Planctomycetes* (*P* = 0.001) in comparison to both the control and monensin treatments. Significant negative correlations were observed between the abundances of *Bacteroides, Fusobacterium*, and *Succinivibrio* genera and methane (*r* > -0.71; *P* ≤ 0.001). The abundance of *Fibrobacter* genera and total SCFA (*r* = 0.86), acetate (*r* = 0.75), and valerate (*r* = -0.51; *P* < 0.001) correlated positively. These results suggest that *S. striata* supplementation at 80 mg g^-1^ DM inclusion, similar to monensin, supports rumen fermentation, lowers methane and ammonia production. However, *S. striata* supported rumen fermentation toward higher total SCFA and propionate production, while unlike monensin still supported a diverse rumen microbiome and an increase in cellulolytic bacteria such as *Fibrobacter*.

## Introduction

Rumen microbiota are essential for the host, providing short-chain fatty acids (SCFA), which make up 75% of the total metabolizable energy for ruminants (Siciliano-Jones and Murphy, 1989; Bergman, 1990). In dairy cattle, of the major SCFA, propionate is critical for gluconeogenesis (den Besten et al., 2013) and therefore has particular importance for meeting the high glucose demands of lactation. In addition, the production of propionate is an electron-accepting pathway and serves as a H^+^ sink, lowering the substrate availability for methanogenesis (den Besten et al., 2013). The production of methane not only has high global warming potential, but also reduces feed energy efficiency in ruminants (Johnson and Johnson, 1995). Therefore, ruminant nutritionists have been working for decades to develop dietary strategies capable of modulating both the rumen fermentation profile to increase propionate production for high producing cattle and reducing methane production (McAllister and Newbold, 2008; Hristov et al., 2013).

In this regard, monensin, an ionophore widely used as a growth promoter (Shen et al., 2017), has been previously shown to inhibit ruminal methanogenesis and promote the production of propionate (Green et al., 1999; Duffield et al., 2008). However, because of the increasing concerns about antibiotic resistance and since the ban of antibiotics in the European Union (Broudiscou et al., 2000; Shen et al., 2017), considerable efforts have been devoted to investigating the potential use of plant phytochemicals such as essential oils, saponins and tannins as alternatives to antibiotics in ruminant nutrition (Śliwiński et al., 2002; Bodas et al., 2012). *Scrophularia striata* is a herbaceous flowering plant, commonly known as figwort, which is often used in traditional folk remedies against infections (Monsef-Esfahani et al., 2010; Mahboubi et al., 2013). Previous research has shown that the ethanolic extract of *S. striata* has antimicrobial activity against *Staphylococcus aureus, Eschericia coli, Enterococcus faecalis*, and Newcastle disease (Bahrami and Valadi, 2010; Bahrami, 2011). However, the effects of *S. striata* extract on the anaerobic microbiota of the rumen and the associated fermentation have not been previously reported.

Therefore, this study was designed to evaluate effects of *S. striata* on SCFA production, methane production as well as the diversity and abundance of the rumen microbiome compared to monensin in a semi continuous *in vitro* system. We hypothesized that feeding of *S. striata*, similar to monensin, could alter the rumen microbiome leading to beneficial modifications to rumen fermentation including increased propionate production and an inhibition of ruminal methane production.

## Materials and Methods

### Plant Material and Extraction

*Scrophularia striata* was purchased from a local herbalist’s shop in Ilam, a province in the west of Iran and was authenticated as *S. striata* Bioss family (*Scrophulariaceae*) with a voucher specimen being preserved in the pharmacy herbarium (TEH No: 6916) at Tehran University of Medical Science, Tehran, Iran. Whole plants containing stem and seed were ground in a centrifugal mill with 0.5 mm screen. Ground plant material was mixed with 600 ml L^-1^ aqueous ethanol solution at the ratio 1:25 (w:v) for 1 h in sonicator bath with cooling system (Sonorex RK 156H, Bandelin, Berlin, Germany). The mixture was filtered through filter paper and concentrated in a vacuum at 40°C by a rotary evaporator (Buchi, Flawil, consisting of water bath B-480, Rotovapor R-124, and Vacuum Controller B-72, Switzerland). After ethanol evaporation, the particulate was transferred to beaker flask, frozen at -20°C and then freeze-dried according to previously described methods (Goel et al., 2008).

### Experimental Design, Rusitec Procedure, and Sample Collection

The experiment was designed as a completely randomized design with 2 experimental runs, 4 treatments, and 3 replicates per run, resulting in six independent measurements per treatment. The treatments consisted of either no additives (control), 0.83 mg g^-1^ DM of substrate monensin sodium salt (monensin; Sigma Chemical Co. Ltd.), 40 mg g^-1^ DM (*S. striata*-Low), or 80 mg g^-1^ DM (*S. striata*-High) of dried of *S. striata* extract. Monensin and *S. striata* were dissolved in 1 ml ethanol [90% (v/v), TechniSolv^®^, pure] and an equal volume of ethanol was added to the control treatment group (Cardozo et al., 2005).

All runs were comprised of 12 fermenters and lasted for 10 days, with sampling on the last 5 days of each run based on a 4 day adaptation period for the rumen microbes (Soliva and Hess, 2007). Initial inocula for each experimental run was collected from three non-lactating rumen-fistulated Holstein cows, housed at the Dairy Research Station of the University of Veterinary Medicine Vienna (Pottenstein, Austria). The use of donor cows was approved by the institutional ethics committee of the University of Veterinary Medicnie (Vetmeduni). Donor cattle were fed hay and grass silage *ad libitum* and free access to drinking water. Animals were cared for according to the Austrian guidelines for animal welfare (Federal Ministry of Health, 2004). Rumen contents of the three cows were mixed in equal parts, the liquor was first filtered through four layers of medical gauze (1-mm pore size) before adding to the fermenters and the solid digesta was filled (∼60 g) into nylon bags and placed into each fermenter on the first day of each experimental run according to the previously published methodology (Klevenhusen et al., 2015). In brief, each fermenter was filled with 100 ml artificial saliva and 600 ml rumen fluid on the first day of each experimental run (McDougall, 1948). New substrate was provided to each fermenter daily, based on a 50:50 forage-to-concentrate dairy ration (**Supplementary Table S1**) using nylon bags (140 mm × 70 mm, 150-μm pore size, Linker Industrie–Technik, Kassel, Germany). After 24 h of incubation, the bags containing solid digesta were replaced with fresh bags containing the experimental diet. Accordingly, every feed bag was incubated for 48 h, and then replaced. Artificial saliva buffer was continuously infused into each vessel (357 ± 19.2 ml/d) through a 12 channel peristaltic pump (Model ISM 932D, Ismatec, Index Health and science GmbH, Wertheim, Germany) in accordance with Soliva and Hess (2007). Feed additives were pipetted into the fermenter fluid every day at the time of feeding (0900 h) starting on day 0. After each feeding procedure the system was flushed with N_2_ gas for 3 min to maintain anaerobic condition inside the vessels. Daily effluent and fermentation gasses were collected in a 1 L volumetric flask located in an ice-filled box and reusable gas-tight aluminum bags (TECOBAG 8L, Tesseraux ContainerGmbH, Burstadt, Germany), respectively. On sampling days, directly before feed bag exchange, fermenter fluid samples were collected using a syringe equipped with a plastic tube. Part of the fluid samples were immediately analyzed for rumen fermentation parameters and aliquots were stored in separate tubes at -20°C for SCFA and ammonia measurements. Additional fluid samples were snap frozen in liquid nitrogen and stored at -80°C for DNA extraction. The feed residue bags were hand-washed with running water until the water was clear and kept at -20°C for chemical composition analysis.

### Sample Analysis

Immediately after sampling pH and redox potential (seven Multi^TM^, Mettler-Toledo GmbH, Schwerzenbach, Switzerland) of the rumen fermenter liquid were measured. Fermentation gasses were also measured every 24 h during the sampling period. Gas volume was determined using the water displacement technique (Soliva and Hess, 2007), and the concentration of CH_4_ was measured using a portable infrared detector (ATEX biogas Monitor Check BM 2000, Ansyco, Karlsruhe, Germany). In brief, the biogas monitor is attached to the gas collection bag after a 24 h collection period, the sample is measured through pumping of gas into the monitor for 30 s to provide a consistent reading as per the manufacturer’s instructions.

Concentration and composition of SCFA (acetate, propionate, *n*-butyrate, isobutyrate, *n*-valerate, isovalerate, and caproate) were analyzed by GC, as described previously (Qumar et al., 2016). Briefly, incubation fluid samples were thawed and then centrifuged at 20,000 × *g* for 25 min at 4°C. The supernatant (0.6 mL) was transferred into a fresh tube and 0.2 mL of HCl (1.8 mol L^-1^) was added, followed by 0.2 mL of the internal standard (4-methylvaleric acid, Sigma-Aldrich Co. LLC., St. Louis, MI, United States). After centrifugation of the mixture at 20,000 × *g* for 25 min at 4°C, the clear supernatant was transferred into the GC vial. Analysis of SCFA concentrations was conducted via a gas chromatography apparatus (GC Model 8060 MS 172 DPFC, No.: 950713, Fisons, Rodano, Italy) which was equipped with a flame-ionization detector and a 30 m × 0.53 mm ID × 0.53 μm df capillary column (Trace TR Wax, Thermo Fisher Scientific, Waltham, MA, United States). Injector and detector were at temperatures of 170°C and 190°C, respectively. Helium was used as carrier gas with a flow rate of 6 mL min^-1^. Stratos Software (Stratos Version 4.5.0.0, Polymer Laboratories, Church Stretton, Shropshire, United Kingdom) was utilized for generation and evaluation of chromatograms.

The concentration of ammonia was determined using the indophenol reaction (Weatherburn, 1967). The fermenter fluid samples were thawed at room temperature and subsequently centrifuged at 15,115 × *g* for 10 min. The clear supernatant was diluted with distilled water to obtain a concentration within the standard calibration curve. Ammonia and phenol were oxidized by sodium hydroxide in the presence of sodium nitroprusside and dichloroisocyanuric acid. The absorbance was measured at 655 nm using U3000 Spectrophotometer (INULA GmbH, Vienna, Austria) after 90 min of reaction.

Analysis of the residual feeds after 48 h of incubation and the original feed samples were conducted in duplicate. Prior to analysis, samples were oven-dried at 65°C for 48 h and ground using through a 0.75 mm sieve (VDLUFA, 2007). The DM of feeds and feed residues was measured after oven drying at 104°C for 24 h and ash was analyzed by combustion of samples over night at 580°C. Crude protein was measured by Kjeldahl method (VDLUFA, 2007). Neutral detergent fiber (aNDFom; Udén et al., 2005) and acid detergent fiber (ADFom) contents were analyzed using Fibretherm FT12 (C. Gerhardt GMbH and Co. KG, Konigswinter, Germany) based on VDLUFA (2007). Heat stable α-amylase used in the NDF procedure and all fiber fractions were expressed exclusive residual ash. Disappearance of nutrients as a measure of degradability was calculated from the difference between content of nutrients in the diet before and after 48 h incubation in the Rusitec system.

### DNA Extraction and qPCR

Frozen rumen fluid samples were thawed on ice, vortexed shortly to mix the sample, and then samples from d 5 to 10 were pooled per fermenter per run (*n* = 6 per treatment). From the pooled sample, 1 mL subsample was used for DNA isolation. DNA isolation was performed using the QIAamp Fast DNA Stool Mini Kit (QIAGEN, Hilden, Germany) and samples were mixed with 1 mL of InhibitEX buffer provided in the kit and heated at 95°C for 5 min to ensure proper lysis of bacteria. After heating, additional enzymatic (mutanolysin, 2.5 U μL^-1^; lysozym, 100 mg mL^-1^; proteinase K, 20 mg mL^-1^) and mechanical lysis (0.4 g sterile ceramic beads, diameter 1.4 mm) using a bead-beater (FastPrep-24 5G, MP Biomedicals, LLC, Santa Ana, CA, United States) was performed to disrupt bacterial cells according to previously published procedures (Kong et al., 2010). This was followed by chemical removal of cell debris and PCR inhibitors by centrifugation and supernatants were transferred to fresh tubes for column based isolation of total genomic DNA using the QIAcube robotic workstation. Total genomic DNA was eluted in 200 μL of low-salt buffer. The isolated DNA concentration was determined by a Qubit 2.0 Fluorometer (Life Technologies, Carlsbad, CA, United States) using the Qubit dsDNA HS Assay Kit (Life Technologies) and stored at -20°C until further analysis.

### Quantitative PCR Analysis

Using qPCR, the abundance of Protozoa, Bacteria, Archaea, and Fungi present in Rusitec samples were analyzed on a Stratagene Mx3000P real-time PCR System (Agilent Technologies, Santa Clara, CA, United States) using established primer pairs (**Table 1**). DNA samples and negative controls were assayed in duplicate in a 20 μL reaction mixture containing 10 μL the Fast-Plus EvaGreen Master Mix with Low ROX (Biotium, Hayward, CA, United States), 1 μL of each primer (Protozoa 400 nM; Bacteria 100 nM; Archaea and Fungi 200 nM), 7 μL of nuclease-free water and 1 μL DNA template (4 ng). The amplification program included an initial denaturation step at 95°C for 10 min, followed by 40 cycles of 95°C for 10 s, primer annealing at 60°C for 30 s, and elongation at 72°C for 30 s. Fluorescence was measured at the last step of each cycle. Melting curve analysis was performed to determine the specificity of the amplification. The dissociation of PCR products was monitored by slow heating with an increment of 0.1°C s^-1^ from 55 to 95°C, with fluorescence measurement at 0.1°C intervals. Standard curves for all primers were generated using 10-fold serial dilutions (10^8^–10^3^ molecules μL^-1^) of the purified and quantified PCR products generated by standard PCR using DNA from the pooled rumen fluid from both runs of the present experiment. The PCR efficiency was calculated: E = 10^(-1/slope)^-1 (**Table 1**). Results were analyzed using the associated software (Stratagene MxPro, QPCR Software, version 2.00).

**Table 1:** Primers for kingdom level analysis using quantitative polymerase chain reaction analysis.

Primer	Primer sequence 5′-3^′1^	Product size (bp)^2^	Tm^3^ (°C)	Primer efficiency	Reference
Archaea 16S rRNA	f:CCGGAGATGGAACCTGAGAC	160	60	0.97	Zhou and Hernandez-Sanabria, 2009
	r:CGGTCTTGCCCAGCTCTTATTC				
Universal bacteria 16S rRNA	f:CCTACGGGAGGCAGCAG	189	55	1.02	Muyzer et al., 1993
	r:ATTACCGCGGCTGCTGG				
Total protozoa 18S rRNA	f:GCTTTCGWTGGTAGTGTATT	233	60	1.04	Sylvester et al., 2004
	r:CTTGCCCTCYAATCGTWCT				
Anaerobic fungi ITS1	f:GAGGAAGTAAAAGTCGTAACAAGGTTTC	110-115	60	0.99	Denman and McSweeney, 2006
	r:CAAATTCACAAAGGGTAGGATGATT				

*^*1*^f: forward primer, r: reverse primer. ^*2*^Product size in basepairs (bp). ^*3*^Tm: melting temperature in Celcius.*

### Sequencing, Sequence Processing, and Bioinformatics Analysis

One 40 μL aliquot of each DNA sample was sent for amplicon sequencing using a MiSeq Illumina sequencing platform and paired-end technology (Microsynth AG, Balach, Switzerland). Sequencing targeted the V3–V5 hypervariable region of the 16S rRNA gene using the primer set 357F-HMP (5′-CCTACGGGAGGCAGCAG-3′) and 926R-HMP (5′-CCGTCAATTCMTTTRAGT-3′) to produce an amplicon size of ∼523 bp (Peterson et al., 2009). In brief, libraries were constructed by ligating sequencing adapters and indices onto purified PCR products (Nextera XT Sample Preparation Kit, Illumina, San Diego, CA, United States) according to the recommendations of the manufacturer. Equimolar amounts of each of the libraries were pooled together and sequenced on an Illumina MiSeq Personal Sequencer and the resulting paired ends were stitched together by Microsynth (Balach, Switzerland) resulting in a total of 3.8 million unfiltered reads with an average of 503 nt in length. Sequence quality control was performed using the QIIME pipeline (Caporaso et al., 2010). Sequences were first quality filtered following (Q = 20; 27) and then screened and filtered for chimeras using USEARCH and its database (USEARCH v8.1; Bokulich et al., 2013). Clustering and alignment were performed using UCLUST (Edgar, 2010) and OTUs were defined using PyNAST (Caporaso et al., 2010) and the SILVA database (v123; accessed July 19, 2017; Quast et al., 2012; Yilmaz et al., 2013). The degree of similarity between sequences was defined as 97% to obtain OTU. Any OTUs which clustered with less than 10 reads were manually removed. A total of 969,155 sequences (mean sequences per sample 38,504) clustered into 2,727 OTUs for further analysis. All OTUs with a relative abundance greater than 0.1% (135 OTUs) were taken in consideration for statistical analysis and statistically derived means for relative abundance were graphed in excel. For calculation of the non-parametric species richness estimators Chao1 and the observed OTUs per sample were used. Beta diversity analysis was performed using weighted Unifrac dissimilarity metrics and the principal coordinate analysis (PCoA) plotting in QIIME with rarefaction at 20,133 sequences, based on the lowest number of sequences in a sample. Sequencing data are available in BioProject SRA database under the accession number SRP140677.

### Statistical Analyses

Analysis of variance was conducted using the PROC MIXED procedure of SAS (Version 9.2, SAS Institute, Cary, NC, United States). Treatments were considered as fixed effects, while experimental run and fermenter within run were defined as random effects. Degrees of freedom estimation was done using Kenward-Rogers. All values were reported as least squares means. Comparison between the control and treatments was carried with Dunnett–Hsu. Differences were declared as significant at *P* ≤ 0.05. Correlation analysis using Pearsons correlation coefficient was performed using the PROC CORR procedure of SAS. Following Hinkle et al. (2003), the *r* was interpreted as follows: 0.00–0.30 as negligible; 0.30–0.50 as low; 0.50–0.70 as moderate; 0.70–0.90 as high, and 0.90–1.00 as substantial.

## Results

### Rumen Fermentation Profile, Methane Production, and Nutrient Degradability

The pH in the fermenters remained constant throughout both experimental runs (**Supplementary Figure S1** and **Table 2**). However, redox potential in the fermenters was lower for all treatments when compared to the control (*P* = 0.007). Analysis showed that the ammonia concentration decreased (*P* < 0.001) with monensin and *S. striata* supplementation. The extent of ammonia reduction was 35, 26, and 52%, respectively, by monensin, *S. striata*-Low and *S. striata*-High treatments. Total SCFA production was decreased by monensin, while *S. striata* supplementation increased it compared with control (*P* = 0.001). All individual SCFA were affected by supplementation (**Table 2**). Monensin decreased acetate production by 9%, whereas *S. striata* had no effect in comparison with control. Propionate was increased by 186, 83, and 35% by feeding monensin, *S. striata*-High and *S. striata*-Low, respectively. As a result, monensin and *S. striata*-High had a lower acetate to propionate ratio (A:P) in comparison to control (*P* < 0.001). Supplementation decreased iso-butyrate concentrations in comparison to control (*P* < 0.001). Butyrate was decreased by monensin and *S. striata*-High (*P* < 0.001) in comparison to control. In contrast, the addition of *S. striata*-Low increased the concentration of iso-valerate above that of the control group (**Table 2**). Monensin decreased the proportion of iso-valerate to 8.1% of the control concentration and increased of *n*-valerate by 97% (*P* < 0.001). Supplementation with monensin, *S. striata*-Low and *S. striata*-High caused a drop in methane production by 67, 29, and 36%, respectively (*P* < 0.003). The DM, OM, and ADF degradation were not affected by supplementation (**Table 2**). However, *S. striata*-High decreased CP degradation (*P* = 0.03) and all additives decreased NDF degradability (*P* = 0.02) compared to the control group.

**Table 2:** Effect of supplementation of *Scrophularia striata* extract and monensin on rumen fermentation profile and nutrient degradation.

Parameter	Treatments^1^				SEM^2^	*P*-value
	Control	Monensin	*S. striata*-Low	*S. striata*-High		
pH	6.58	6.57	6.56	6.56	0.033	0.95
Redox (mv)	–263.0^a^	–224.7^b^	–214.6^a^	–233.2^b^	7.89	0.01
NH_3_ (mmol L^-1^)	8.65^a^	5.56^b^	6.39^b^	4.14^b^	0.472	<0.001
CH_4_ (ml day^-1^)	119^a^	38.7^b^	84.5^b^	75.9^b^	4.46	<0.001
CH_4_ reduction (% of Control)	–	67.2	28.8	35.7	3.50	0.003
Total SCFA (mmol d^-1^)	35.3^a^	32.7^b^	39.4^b^	38.5^b^	0.69	<0.001
Individual SCFA (mmol d^-1^)						
Acetate (A)	17.4^a^	14.7^b^	19.5^b^	19.5^b^	0.675	<0.001
Propionate (P)	4.2^a^	11.2^b^	6.4^b^	8.4^b^	0.50	<0.001
Isobutyrate	0.20^a^	0.10^b^	0.18^b^	0.10^b^	0.001	<0.001
*n*-Butyrate	4.8^a^	1.9^b^	4.2^a^	3.7^b^	0.72	<0.001
Isovalerate	3.30^a^	0.27^b^	4.85^b^	2.88^b^	0.338	<0.001
*n*-Valerate	1.87^a^	3.70^b^	2.23^a^	2.47^b^	0.264	<0.001
Caproate	3.55^a^	0.92^b^	2.07^b^	1.37^b^	0.129	<0.001
A:P	4.34^a^	1.33^b^	3.18^b^	2.36^b^	0.418	<0.001
Nutrient degradation (%)^3^						
Dry matter	62.5	61.4	61.0	60.1	1.00	0.16
Organic matter	59.2	58.0	57.7	56.7	1.04	0.29
Crude protein	69.5^a^	68.1^a^	66.9^a^	65.2^b^	1.03	0.03
Neutral detergent fiber	24.0^a^	19.1^b^	20.4^a^	18.1^b^	1.12	0.02
Acid detergent fiber	17.0	14.1	14.8	14.3	1.86	0.11

*^*1*^Control: no additive; Monensin, contained 0.83 mg g^-*1*^ of DM of substrate monensin sodium salt; S. striata-Low and S. striata-High contained 40 and 80 mg g^-*1*^ of DM of substrate dried plant extract, respectively. ^*2*^SEM: standard error of the mean. ^*3*^Methods of analysis are based on the VDLUFA (2007) methods of feed composition analysis. ^*a,b*^Means within the same row with different superscripts differ to the Control treatment at *P* < 0.05.*

### Quantitative Analysis at the Kingdom Level of Taxonomy

**FIGURE 1:**
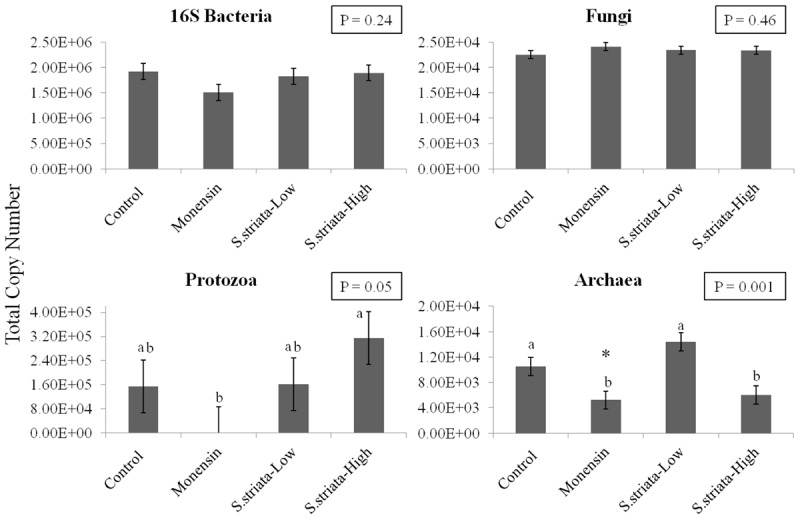
Effect of supplementation of *Scrophularia striata* extract, in two doses (*S. striata*-Low and *S. striata*-High), and monensin on copy number of Bacteria (16S rRNA), Fungi (ITS Gene 1), Protozoa (18S rRNA), and Archaea (16S rRNA) analyzed by qPCR. Data is presented in total copy numbers of the target gene. ^∗^In tendency lower than control (*P* = 0.08). ^a,b^Treatments with different letters differ significantly to the Control *P* < 0.05.

Results of qPCR analysis are presented in **Figure 1**. Supplementation did not affect the abundance of total Bacteria and Fungi populations compared to control. Monensin remarkably lowered the gene copy number of Protozoa (1.00 × 10^2^), though it was significant only when compared to *S. striata*-High group (3.15 × 10^5^; *P* = 0.05). Copy numbers of total Archaea were lower with *S. striata*-High and monensin compared to *S. striata*-Low (*P* = 0.001), and tended to be lower with monensin compared to control (*P* = 0.08).

### Alpha and Beta Microbial Diversity

**FIGURE 2:**
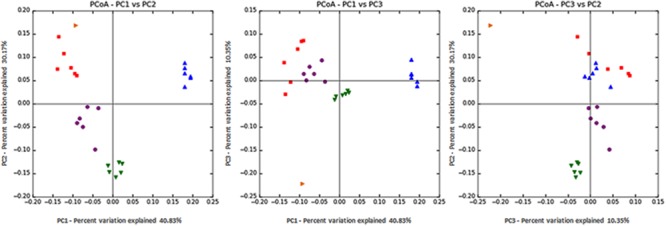
Principal coordinate analysis (PCoA) of 16S rRNA using the weighted Unifrac distance metric (PC1 = 40.8%, PC2 = 30.2%, and PC3 = 10.3%). Orange: original rumen fluid sample taken from cows, Red: control, Blue: monensin, Purple: *S. striata*-Low, and Green: *S. striata*-High. *S. striata*: *Scrophularia striata* extract.

Statistical analysis of both the intra- (alpha diversity; **Table 3**) and inter- (beta diversity; **Figure 2**) sample diversity was performed. Despite no differences in the total numbers of sequences found within a sample (*P* = 0.91), there were differences between treatments in the number of OTUs (*P* < 0.001) and the Chao1 index (*P* < 0.001). For both indices, the diversity within a sample was highest in the control, followed by *S. striata*-Low, *S. striata*-High, and monensin showed the least microbial diversity. PCoA showed a clear clustering of microbial communities for each treatment group. Principle component 1 described 40.8% of the diversity between samples between original rumen fluid and experimental treatments, with the monensin treatment clustering independently (**Figure 2**). Principle component 2 represented 30.2% of the diversity with a separation of the *S. striata* samples from the control and monensin groups. Principle component 3, representing 10.3% of the variation between samples, was related to the original pooled rumen fluid sample.

**Table 3:** Effect of supplementation of *Scrophularia striata* extract and monensin on number of sequences, observed operational taxonomic units (OTU) and the Chao1 index.

	Treatments^1^					
Parameter	Control	Monensin	*S. striata*-Low	*S. striata*-High	SEM^2^	*P*-value
Number of sequences	38560	39202	36323	36470	5141.0	0.91
Observed OTUs	1884^a^	726^b^	1319^b^	1003^b^	103.1	<0.001
Chao1 index	3536^a^	1655^b^	2727^b^	2068^b^	137.9	<0.001

*^*1*^Control: no additive; monensin: monensin, contained 0.83 mg g^-*1*^ of DM of substrate monensin sodium salt; S. striata-Low and S. striata-High contained 40 and 80 mg g^-*1*^ of DM of substrate dried plant extract, respectively. ^*2*^SEM: standard error of the mean. ^*a,b*^Means within the same row with different superscripts differ to the Control treatment at *P* < 0.05.*

### 16S rRNA Taxonomy

**FIGURE 3:**
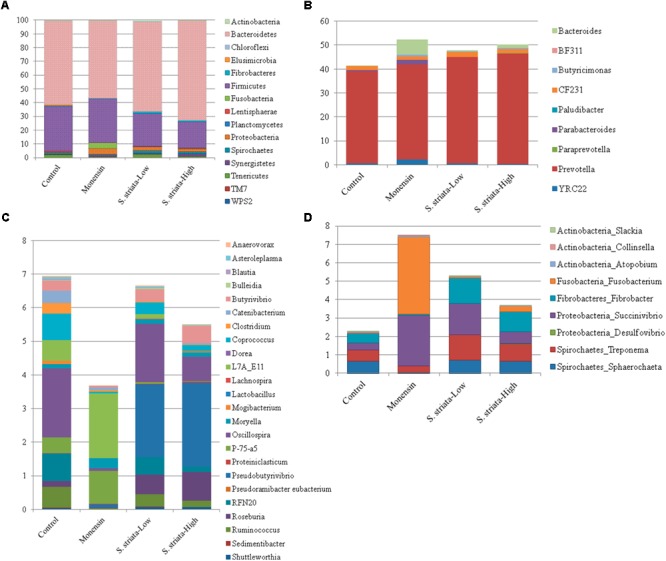
Mean relative abundance of 16S rRNA sequences identified to 97% with the SILVA128 database at the phyla level **(A)** and genera level for *Bacteroidetes* **(B)**, *Firmicutes* **(C)**, and other groups **(D)**. All phyla and genera have a significant effect of supplementation (*P* ≤ 0.05).

**FIGURE 4:**
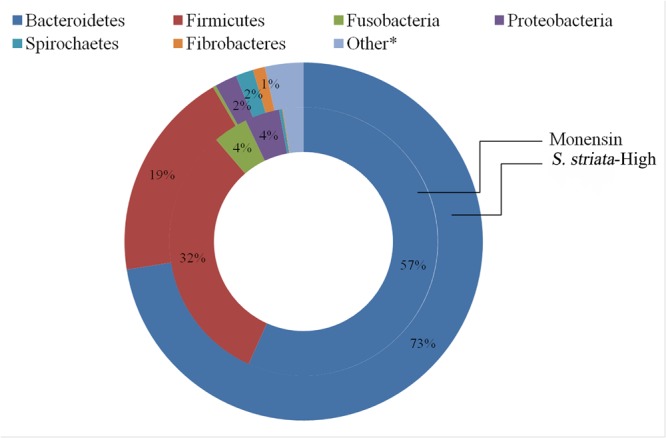
Comparison of the mean relative abundance of phyla between treatment groups supplemented with *Scrophularia striata* extract at 80 mg g^-1^ DM (*S. striata*-High) and those given monensin at 0.83 mg g^-1^ DM. Differences in the *P*-value for least square means between monensin and *S. striata*-High are *Bacteroidetes* (*P* < 0.001), *Firmicutes* (*P* < 0.001), *Fusobacteria* (*P* < 0.001), *Proteobacteria* (*P* = 0.001), *Fibrobacteres* (*P* = 0.001), *Spirochaetes* (*P* = 0.001). Other includes Lentisphaerae (*P* = 0.04), *Planctomycetes* (*P* = 0.001), *Actinobacteria* (*P* = 0.96), *Chloroflexi* (*P* = 0.65), *Elusimicrobia* (*P* = 0.99), *Synergistetes* (*P* = 0.69), *Tenericutes* (*P* = 0.37), *TM7* (*P* = 0.99), *WPS-2* (*P* = 0.28). ^∗^Individual phyla within the “Other” group do not have means which are significantly (*P* < 0.05).

**FIGURE 5:**
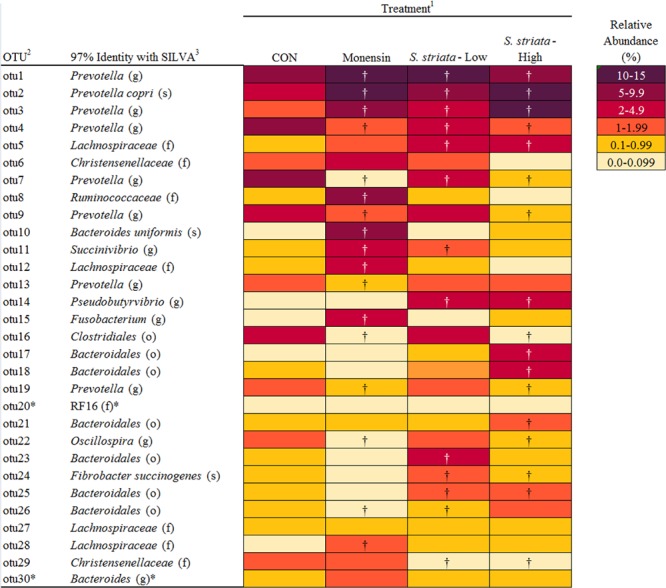
Effect of supplementation of Scrophularia striata extract and monensin on percent relative abundance and taxonomic classification of 30 most abundant operational taxonomic units (OTUs). ^1^Control: no additive; monensin: monensin, contained 0.83 mg g^-1^ of DM of substrate monensin sodium salt; *S. striata*-Low and *S. striata*-High contained 40 and 80 mg g^-1^ of DM of substrate dried plant extract, respectively. ^2^OTUs listed in order of relative abundance. ^3^Name of the closest relative based on 97% similarity in the sequence database at the class (c), order (o), family (f), genus (g), or species (s) level. ^†^Adjusted mean separation based on Dunnett-Hsu shows a significant variation from the Control. ^∗^Means are not significantly different (*P* < 0.05).

In total, 15 phyla, predominated by *Bacteroidetes, Firmicutes*, and *Proteobacteria*, could be identified into 42 genera. All phyla and all genera were significantly affected by treatments (**Figure 3**). Supplementation with *S. striata*-High increased the total *Bacteroidetes* and reduced the *Firmicutes* taxa, whereas monensin increased *Fusobacteria, Synergistetes*, and *Proteobacteria* phyla (**Figure 3A**). The addition of all additives decreased the relative abundance of *Elusimicrobia* and TM7 in comparison to control. *Fusobacterium* and *Firmicutes* were, respectively, 4 and 13% more abundant in the monensin in comparison to the *S. striata*-High supplementation (**Figure 3**). At the genera level, *Prevotella* was the most abundant genus ranging from 38.5% in control to 46.1% in *S. striata*-High and was significantly increased with *S. striata* supplementation. *Bacteroidetes*-associated genera represented the largest proportion of the *in vitro* microbiota despite the fact that samples were taken from the rumen liquid (**Figure 3B**). *Firmicutes* had the largest diversity in identified genera with a significant effect of supplementation (**Figure 3C**). A drastic increase in *Fusobacterium* spp. was found in monensin supplementation compared to the other treatments (**Figure 3D**). Direct comparison of the monensin and *S. striata*-High treatments at the phyla level for significant differences in the least square means showed of the 15 phyla, eight were significantly different between the two treatment groups (**Figure 4**). Of the 30 most abundant OTUs, 29 were significantly affected by the supplementation treatment. *Bacteroidetes* and *Firmicutes* accounted for 16 and 8 of the OTUs, respectively (**Figure 5**). Three OTUs were identified to the species level, *Prevotella copri*-like OTU2, *Bacteroides uniformis*-like OTU10 and *Fibrobacter succinogenes*-like OTU24. In comparison to the control, OTU2 was 4.2-fold and four-fold increased in monensin and *S. striata*-High, respectively (*P* < 0.001). OTU10 accounted for 5.13% of the relative microbial abundance in the monensin supplemented group but was less than 0.6% of the abundance in the other treatments. *F. succinogenes*-like OTU24 was increased in both the *S. striata*-Low (1.05%) and *S. striata*-High (0.87%) supplemented groups compared to both control and monensin.

### Correlations Between Genera and Rumen Fermentation

Several correlations between rumen fermentation parameters and microbial communities significantly altered by the supplementations were found (**Table 4**). *Fusobacterium* spp. showed the greatest number of highly (0.70 < *r* < 0.90) negative correlations, including methane, acetate, butyrate, isovalerate and total SCFA production (*P* < 0.001) and positive correlations to propionate (*P* < 0.001) and valerate (*P* < 0.001). Similarly, *Bacteroides* (phyla *Bacteroidetes*) and *Succinivibro* (phyla *Proteobacteria*) genera also showed an inverse relationship to methane production (*r* = -0.76 and *r* = -0.71, respectively) and a positive correlation to valerate concentrations *in vitro* (*P* < 0.001). Furthermore, *Bacteroides* spp. was also highly correlated with decreases in butyrate and isovalerate, similar to *Fusobacterium* spp. *Succinivibrio* showed similar correlations as *Bacteroides* (**Table 4**). The strongest correlation was that of *Fibrobacter* spp. with total SCFA concentrations (*r* = 0.86; *P* < 0.001), which was supported by additional positive correlations with acetate and isovalerate concentrations. *Lachnospira* spp. showed a highly negative correlation to ammonia concentrations (*P* < 0.001).

**Table 4:** Pearson correlation coefficients (r) between rumen fermentation parameters and genera significantly impacted by supplementation.

	Genera									
Rumen fermentation parameters	*Bacteroides*	*Butyrivibrio*	*Clostridium*	*Fibrobacter*	*Fusobacterium*	*Lachnospira*	*Prevotella*	*Ruminococcus*	*Succinivibrio*	
Methane	–0.76	0.36	0.65	0.34	–0.76	–0.11	–0.2	0.8	–0.71	
Ammonia	–0.32	–0.23	0.71	–0.24	–0.23	–0.71	–0.47	0.75	–0.35	
Acetate	–0.66	0.72	–0.17	0.75	–0.74	0.53	0.33	0.2	–0.41	***P*-value**
Propionate	0.74	–0.39	–0.47	–0.33	0.74	0.13	0.1	–0.78	0.64	>0.05
Isobutyrate	–0.45	0.05	0.77	–0.02	–0.41	–0.51	–0.51	0.83	–0.45	0.05–0.02
Butyrate	–0.73	0.44	0.73	0.47	–0.74	–0.03	–0.31	0.67	–0.58	0.01–0.001
Isovalerate	–0.82	0.56	0.26	0.79	–0.84	0.08	0.06	0.56	–0.46	<0.001
Valerate	0.83	–0.42	–0.53	–0.51	0.82	–0.02	0.04	–0.67	0.72	
Total SCFA^1^	–0.67	0.72	0.06	0.86	–0.74	0.46	0.12	0.22	–0.32	

*^*1*^SCFA: short chain fatty acids.*

## Discussion

We hypothesized that the supplementation of *S. striata*, a plant with known antimicrobial properties (Monsef-Esfahani et al., 2010; Mahboubi et al., 2013), would elicit similar propionigenic and methano-static effects to the ionophore monensin as a result of changes in the rumen microbiome. The most important findings of this study were a decrease in methane production, an enhanced propionate proportion and a higher total concentration of SCFA as a result of *S. striata* supplementation. The increasing effect of *S. striata* on propionate concentration, while decreasing effect on methane and ammonia production, resembles the effects of monensin. However, changes to the rumen microbiome as a result of *S. striata* supplementation were less dramatic than those seen under monensin supplementation.

In the current study, the degradation of aNDFom and CP decreased with *S. striata*-High supplementation *in vitro*. This agrees with previously reported data showing lower degradation of DM by addition of some flavonoid-rich plant extracts and pure flavonid compounds (Oskoueian et al., 2013; Kim et al., 2015). Previous studies have also reported reductions in DM and NDF digestibility with monensin (Poos et al., 1979; Anassori et al., 2012). However, in this study, *S. striata*-High supplementation had a similar effect on degradation as monensin with the exception of CP. This discrepancy among the different studies may be related to several factors such as diet composition, the period of adaptation to the product, the time when the samples were collected, the method of analysis used and the type and concentrations of the feed additives (Bodas et al., 2012). Ionophores are also generally microbial inhibitors and reduce both cellulose digestion and microbial yield (Demeyer and Van Nevel, 1979). Therefore, the effect of supplementation was assumed to be associated with a decrease in bacterial abundance. However, bacterial abundance as measured by copy number and total number of sequences was not affected by supplementation with either monensin or *S. striata*. There is evidence showing that improved feed efficiency is correlated with lower richness in the rumen microbiota (Shabat et al., 2016). Therefore, a possible reason for improved feed efficiency in previous *in vivo* studies with monensin might have been due to lower diversity of the microbiome. As a similar reduction in microbial diversity was observed with *S. striata* supplementation, it can be inferred that *S. striata* would also improve feed efficiency. In line with this, we found that *S. striata* improved efficiency of fermentation (mM SCFA per unit DM degraded).

In the present study, both monensin and *S. striata* additions caused an increase in propionate concentration, resulting in a decrease in the ratio of acetate to propionate. Effect of *S. striata* on decreasing acetate to propionate ratio confirmed results of a previous study reporting that feeding flavonoids compounds increased propionate and decreased the acetate to propionate ratio in dairy cows fed a high concentrate diet (Balcells et al., 2012). These results are also consistent with those of previous *in vitro* studies (Callaway et al., 1997; Shen et al., 2016, 2017). Ionophores work by inhibiting hydrogen producers such as *Ruminococcus* and *Butyrivibrio* and thereby favor propionate and succinate producers (Schären et al., 2017; Shen et al., 2017). The suppression of hydrogen transport diverts carbon away from acetate production toward propionate and increases irreversible acetate loss in the synthesis of butyrate. This same mode of action is hypothesized for *S. striata* based on its antimicrobial properties. In this study, monensin decreased both *Butyrivibrio* and *Ruminococcus* species (butyrate and acetate producers, respectively) and increased the total populations of *Bacteroides, Fusobacterium*, and *Succinivibrio*, which are recognized as major propionate producers (Russell, 2002; Puniya et al., 2015). However, supplementation with *S. striata*, despite decreasing methane production did not show the same increases in *Bacteroides, Fusobacterium*, and *Succinivibrio* and only minor decreases in *Ruminococcus*. In contrast, *S. striata* supplementation increased the relative abundance of *Fibrobacter spp.*, of which the largest family member is *F. succinogenes* (Jeyanathan et al., 2014). *F. succinogenes* similar to *Succinivibrio* (Pope et al., 2011) produces succinate as its principal fermentation end product, while other rumen microbes (e.g., *Selenomonas ruminantium*; Scheifinger and Wolin, 1973) produce propionate via the succinate pathway (Shen et al., 2017). While in this study *Fibrobacter* spp. was significantly increased in the *S. striata* treatments, lactate-producing microbes such as *Selenomonas* were not found to be significantly affected by treatments, indicating that other microbes are potentially responsible for the production of propionate in the *S. striata* supplemented groups. One possibility for the differences between groups is the changes in the *Bacteroides* group, which was significantly increased in the *S. striata* supplementation and is predominated by the genus *Prevotella* spp. *Prevotella* spp. is a well known group of bacteria with extremely diverse metabolic capabilities (Bekele et al., 2010). It is possible that a member of this group is able to convert succinate produced by the *Fibrobacter* spp. to propionate, explaining the variation between the monensin and *S. striata* groups. Interestingly, the total number of *Butyrivibrio* also increased with increasing dosages of *S. striata*, which is in direct contrast to the effects of monensin supplemented groups found in this experiment. Similar to a previous study (Shen et al., 2017), we observed that the relative abundance of *Butyrivibrio* and *Pseudobutyrivibrio* in the *S. striata* treatments was significantly higher than in the monensin treatment. *B. fibrisolvens* (Stewart et al., 1997) and *Pseudobutyrivibrio* spp. (Kopečný et al., 2003) are important butyrate-producing species in the rumen and their presence may explain the higher butyrate levels in the *S. striata* groups compared to monensin group. There is also some evidence of growth promoting effects of phenolic compounds on several pure cultures of cellulolytic and non-cellulolytic bacteria resulting in enhanced acetate production *in vitro* (Borneman et al., 1986). In particular, the cellulolytic bacteria *Treponema* and *Fibrobacter* increased by *S. striata* in the current study, which could explain comparable acetate levels to the control. Accordingly, the abundance of *Fibrobacter* positively correlated with acetate concentration. The positive influence of *S. striata* on total SCFA production is potentially of great benefit to performance animals in order to meet the increased energy requirements of growth and production.

The potential of plant secondary metabolites to affect methane production has varied extensively ranging from -30 to +30% in a screening study with 450 plant species (Bodas et al., 2008). In the present study, the extent of methane reduction was, respectively, 29–36% in *S. striata*-Low and *S. striata*-High which, based on a study that reviewed enteric methane mitigation options, falls within the range of medium to high decrement (Hristov et al., 2013). *Scrophularia* extract supplemented treatments showed a decreased overall abundance of *Firmicutes*, which are important ruminal H_2_-producing microorganisms (Shen et al., 2017), whereas monensin supplementation showed no effect. These results contradict previous research which demonstrated that monensin can directly inhibit H_2_-producing bacteria, thereby indirectly decreasing methane production (Russell and Houlihan, 2003). Despite differences in microbial abundance between monensin and *S. striata* supplemented groups, both groups had decreases in CH_4_ and increases in propionate productions, with a dose dependant response being seen in the *S. striata* supplementation. However, the mechanism of action on methane production is probably different between monensin and *S. striata*. In contrast to *S. striata*, supplementation with monensin decreased the copy numbers of both Archaea and Protozoa populations. This supports previous research which noted a dose dependant response in Protozoa to monensin (Dennis et al., 1986). Therefore, the antiprotozoal effect of monensin seen in this experiment may have resulted indirectly in the reduction of the methanogenic Archaea due to their attachment to and dependency on Protozoa for hydrogen (Morgavi et al., 2010). In *S. striata*-High supplementation groups, Protozoa copy numbers were significantly increased and yet Archaea numbers were decreased. Since differences were seen in the copy numbers in the *S. striata*-Low supplemented groups for both Protozoa and Archaea, this indicated a dose dependant response in these groups to *S. striata* supplementation. These results again contradict previous research which demonstrated that monensin does not directly inhibit methanogens (Russell and Houlihan, 2003).

Since defaunation decreases rumen ammonia concentration resulting in lower deamination, an increased uptake of ammonia by the bacterial population and a smaller recycling of microbial proteins, low Protozoa counts could explain the decreased ammonia concentrations with monensin. However, in the *S. striata*-High treatment, Protozoa populations were highest and ammonia concentrations lowest. Two major groups of bacteria are responsible for ammonia production in the rumen; bacteria with low activity and high number and those with low numbers and high specific activity (Russell et al., 1991). *Clostridium aminophilum, Clostridium sticklandii* (Russell et al., 1988), and *Peptostreptococcus anaerobius* (McIntosh et al., 2003) have been recognized as important hyper-ammonia producing bacteria. The sensitivity of *C. sticklandii* and *P. anaerobius* to essential oils has been previously reported (McIntosh et al., 2003). Therefore, the lower ammonia production and CP degradation in *S. striata* treatments could be attributed to lower abundance of genus *Clostridium* spp. *Ruminococcus* also showed positive significant correlations with ammonia concentration and their abundances decreased with supplementation of monensin and *S. striata*. Lower ammonia concentrations in *S. striata*-High compared to monensin suggests that a higher dose of *S. striata* had greater impact on ammonia producing species. Inhibition of ruminal protein degradation by *S. striata* plant extract has important implications on improving post ruminal delivery of true protein (Szumacher-Strabel and Cieślak, 2010).

## Conclusion

Addition of hydro-alcoholic extract of *S. striata* to rumen fluid *in vitro* elicited similar propionigenic, and methanostatic effects to monensin and decreased the acetate:propionate ratio. Microbial analysis, however, showed that *S. striata* and monensin have distinctly different impacts on relative abundances of the total populations of Protozoa and Archaea, as well as effects on the diversity and taxonomy of the bacterial population. The potential of high level of *S. striata* to decrease ammonia concentration and protein degradability at a greater extent than monensin is promising as it could be translated to more efficacious utilization of nitrogen by the host animal. However, these promising results necessitate the investigation of using *S. striata* in *in vivo* feeding trials.

## Author Contributions

MBV, FK, and QZ designed the experiments. MBV and FK performed the experiments. RP analyzed and interpreted the data. RP and MBV drafted the paper. All authors read and approved the final version of the manuscript.

## Conflict of Interest Statement

The authors declare that the research was conducted in the absence of any commercial or financial relationships that could be construed as a potential conflict of interest.

## Supplementary Material

FIGURE S1 Mean fermenter pH for each treatment group, on each day of the experimental period, averaged from all runs.

TABLE S1 Ingredients and chemical composition of the concentrate and basal diet used as substrate in the RUSITEC.
