# Supplementary figures and images for: Scrophularia striata Extract Supports Rumen Fermentation and Improves Microbial Diversity in vitro Compared to Monensin
Source: Front Microbiol. 2018 Sep 19;9:2164. doi: 10.3389/fmicb.2018.02164 (PMC6156526; doi:10.3389/fmicb.2018.02164)

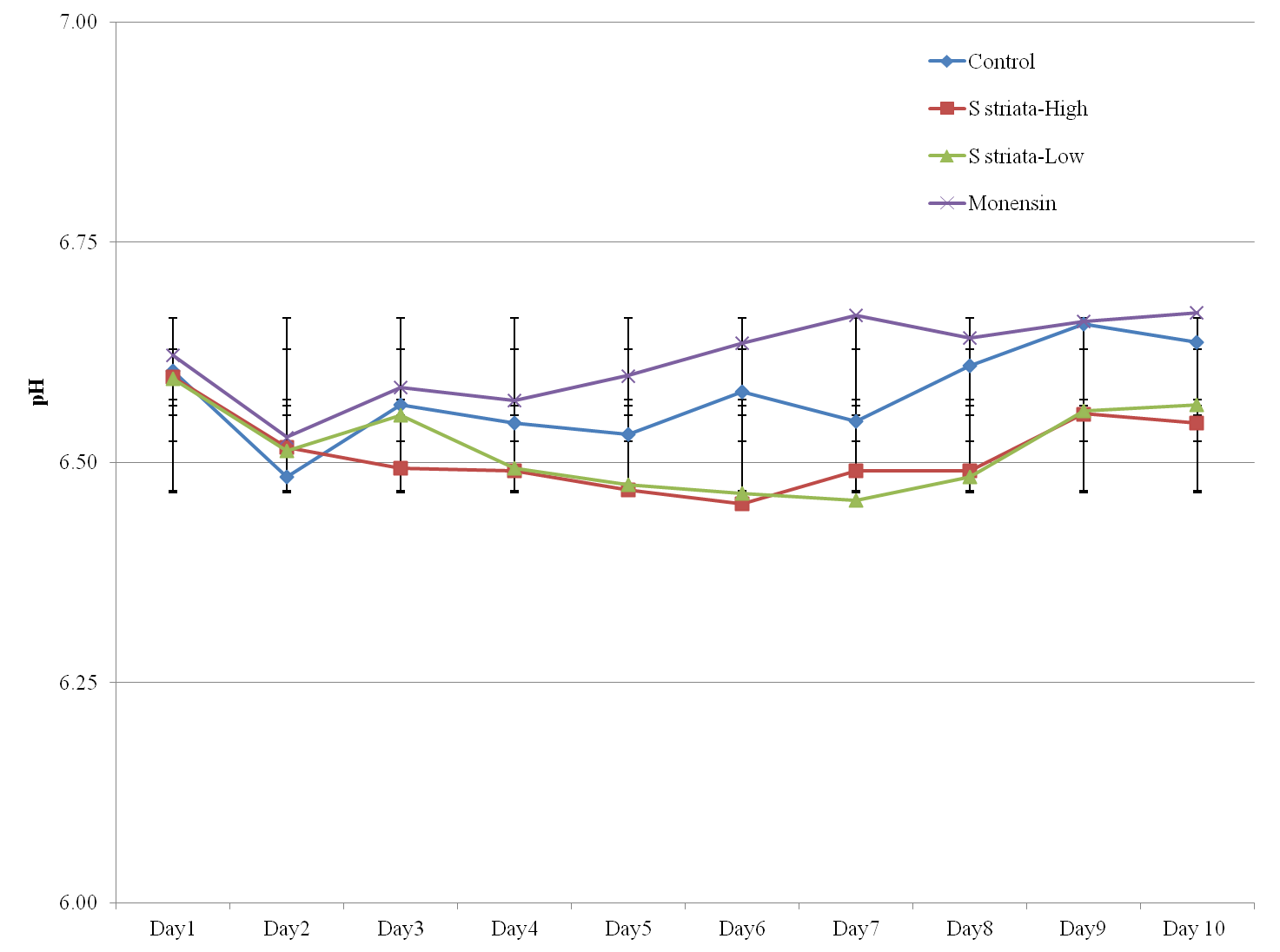

Supplement: FIGURE S1 — Mean fermenter pH for each treatment group, on each day of the experimental period, averaged from all runs. [file Image_1.TIF]
