# Supplementary material for: Scrophularia striata Extract Supports Rumen Fermentation and Improves Microbial Diversity in vitro Compared to Monensin
Source: Front Microbiol. 2018 Sep 19;9:2164. doi: 10.3389/fmicb.2018.02164 (PMC6156526; doi:10.3389/fmicb.2018.02164)
Supplement: TABLE S1 — Ingredients and chemical composition of the concentrate and basal diet used as substrate in the RUSITEC. [file Table_1.docx]

| **Supplementary Table 1.** Ingredients and chemical composition of the concentrate and basal diet used as substrate in the RUSITEC | |
| --- | --- |
| Item | g kg^-1^ dry matter |
| Ingredients |  |
| Grass hay | 500 |
| Concentrate | 500 |
| Concentrate composition | |
| Barley grain | 371 |
| Wheat bran | 225 |
| Corn | 250 |
| Rapeseed meal | 111 |
| Molasse | 20 |
| Calcium carbonate | 15 |
| NaCl | 5 |
| Mineral-vitamin premix^1^ | 3 |
| Chemical composition of the basal diet | |
| Organic matter (OM) | 936 |
| Crude protein (CP) | 167 |
| Neutral detergent fiber (NDF) | 373 |
| Acid detergent fiber (ADF) | 195 |
| ^1^ The vitamin-mineral mix contained per g DM: 60.0 mg Ca; 40.0 mg P; 15 mg Na; 60.0 mg Mg; 1000 IU Vit. A; 150 IU Vit. D3; 4.0 mg Vit. E; 0.29 mg I; 0.04 mg Co; 1.6 mg Cu; 4.2 mg Mn; 6.8 mg Zn; 0.05 mg Se. | |
